# WNT3A‐loaded exosomes enable cartilage repair

**DOI:** 10.1002/jev2.12088

**Published:** 2021-05-19

**Authors:** Bethan L. Thomas, Suzanne E. Eldridge, Babak Nosrati, Mario Alvarez, Anne‐Sophie Thorup, Giovanna Nalesso, Sara Caxaria, Aida Barawi, James G. Nicholson, Mauro Perretti, Carles Gaston‐Massuet, Costantino Pitzalis, Alison Maloney, Adrian Moore, Ray Jupp, Francesco Dell'Accio

**Affiliations:** ^1^ Barts and the London School of Medicine and Dentistry William Harvey Research Institute Queen Mary University of London London UK; ^2^ UCB Pharma Slough Berkshire UK; ^3^ School of Veterinary Medicine University of Surrey Daphne Jackson Road Guildford UK; ^4^ Dipartimento di scienza e tecnologia del farmaco Università degli Studi di Torino Torino Italy

**Keywords:** cartilage, drug delivery, exosomes, joint repair, WNT3a

## Abstract

Cartilage defects repair poorly. Recent genetic studies suggest that WNT3a may contribute to cartilage regeneration, however the dense, avascular cartilage extracellular matrix limits its penetration and signalling to chondrocytes. Extracellular vesicles actively penetrate intact cartilage. This study investigates the effect of delivering WNT3a into large cartilage defects in vivo using exosomes as a delivery vehicle. Exosomes were purified by ultracentrifugation from conditioned medium of either L‐cells overexpressing WNT3a or control un‐transduced L‐cells, and characterized by electron microscopy, nanoparticle tracking analysis and marker profiling. WNT3a loaded on exosomes was quantified by western blotting and functionally characterized in vitro using the SUPER8TOPFlash reporter assay and other established readouts including proliferation and proteoglycan content. In vivo pathway activation was assessed using TCF/Lef:H2B‐GFP reporter mice. Wnt3a loaded exosomes were injected into the knees of mice, in which large osteochondral defects were surgically generated. The degree of repair was histologically scored after 8 weeks. WNT3a was successfully loaded on exosomes and resulted in activation of WNT signalling in vitro. In vivo, recombinant WNT3a failed to activate WNT signalling in cartilage, whereas a single administration of WNT3a loaded exosomes activated canonical WNT signalling for at least one week, and eight weeks later, improved the repair of osteochondral defects. WNT3a assembled on exosomes, is efficiently delivered into cartilage and contributes to the healing of osteochondral defects.

## 1 INTRODUCTION

Cartilage defects are present in 61% of patients undergoing knee arthroscopy (Curl et al., 1997; Hjelle et al., 2002), can be disabling, and have the potential to progress into osteoarthritis (Dell'accio & Vincent, 2010). Cell based approaches to cartilage repair are effective, but, due to the largely autologous nature of the transplanted cells, such procedures are costly and laborious. A number of novel biological agents are being developed which harness and support the endogenous capacity of joint to repair (Sherwood, 2019). One major limitation in delivering such agents (often macromolecules) is the avascular nature of the cartilage tissue and the dense and strongly negatively charged extracellular matrix. Antibodies only penetrate the very superficial layers (Didomenico et al., 2016, 2017). Our recent discovery that extracellular vesicles from neutrophils actively penetrate cartilage, suggests that extracellular vesicles may be ideal vehicles to deliver bioactive molecules to cartilage (Headland et al., 2015).

Extracellular vesicles are a heterogeneous group of double membrane bound vesicles, ranging in size from 30–400 nm, and are synthesized either, through direct shedding from the plasma membrane (microvesicles), or by inward budding within multi‐vesicular bodies (exosomes). They are naturally adapted to deliver their contents to cells, can target different tissues, are relatively stable in biological fluids and can travel over long distances. Understanding their potential and harnessing their targeting ability, would open an abundance of therapeutic opportunities in different disease areas and drug delivery (El Andaloussi et al., 2013).

As a proof of concept we chose to deliver WNT3a in a model of cartilage regeneration, because single nucleotide polymorphisms in the *WNT3a* gene are associated with a reduced regenerative capacity of ear lobe cartilage and because of the striking expression pattern of WNT3a at the cartilage regeneration front (Cheverud et al., 2014).

WNTs are a family of highly conserved signalling molecules that signal through a number of different pathways, including the β‐catenin‐dependent (canonical WNT) pathway, and are known to drive physiological processes including developmental morphogenesis, stem cell maintenance and wound healing (Burgy & Königshoff, 2018; Nusse & Clevers, 2017).

WNT‐β‐catenin signalling is activated in response to joint and cartilage injury (Dell'accio et al., 2008). WNT3a is a strong activator of the WNT‐β‐catenin pathway (Nalesso et al., 2011; Yasuhara et al., 2011). In healthy cartilage, excessive activation of this pathway results in cartilage breakdown (Loughlin et al., 2004; Luyten et al., 2009; Nalesso et al., 2011; Zhu et al., 2009); however following injury, when remodelling and cell expansion is necessary to repair tissue, appropriate and timely WNT3a upregulation was associated with improved repair (Cheverud et al., 2014). WNTs are insoluble molecules that normally adhere to the cell surface through binding to heparansulphate proteoglycans. Some WNT molecules, however, can travel long distances assembled either with a carrier molecule called SWIM, or attached to the surface of exosomes (Gross et al., 2012).

Taking advantage of the capacity of WNT3a to be secreted naturally on the surface of exosomes we tested the hypothesis that exosomes are an effective vehicle to deliver biologically active molecules into cartilage and that exosome facilitated delivery of WNT3a would result in a long lasting protective effect and improve the repair of osteochondral defects in mice.

## 2 METHODS

Patients or the public were not involved in this research.

### 2.1 Isolation and characterization of exosomes

Conditioned medium was generated using serum‐free medium containing ITS supplement (Sigma I3146), as per the manufacturer's instructions from L‐cells stably transfected with WNT3a and control L‐cells (ATCC CRL2647/ CRL2648). Briefly, cells were maintained in complete medium containing DMEM/F12 with 10% foetal bovine serum (FBS). L‐cells containing WNT3a were maintained with 0.4 mg/ml G418 for selection. For generation of conditioned medium, cells at 80–90% confluency were split in a 1:4 ratio into a new flask and cultured for 4 days in DMEM without FBS and containing 1x ITS supplement, (G418 was removed from the L‐cells containing WNT3a). Medium was then removed and replaced with fresh medium for a further 3 days. Both batches of medium were then mixed and passed through a 40μm filter before exosome isolation.

Exosomes were isolated as previously described (Gross et al., 2012). Briefly, conditioned medium was centrifuged sequentially at 300 × *g*, 2000 × *g* and 10,000 × *g* to remove dead cells and debris with the supernatant taken forward to the next step. Exosomes were concentrated by ultra‐centrifugation at 100,000 × *g* for 3 h. The exosome pellet was resuspended in PBS and the ultracentrifugation step repeated to wash the exosome preparation, before re‐suspending the exosome pellet in 200ul of PBS for every 50 ml of starting conditioned medium. Particle concentration was determined using the NanosightTM, NS300TM for nanoparticle tracking analysis. Other validation steps included electron microscopy, western blotting and reporter assay.

### 2.2 Western blotting

For analysis of exosomal preparations, equal volumes or particle concentrations were loaded on Tris‐Glycine polyacrylamide gels, and transferred to nitrocellulose membrane for antibody probing.

### 2.3 Reporter assay

HEK293 cells were maintained in complete medium described above. Cells were plated in monolayer and transfected with SUPER8XTOPFlash TCF/LEF ‐ firefly luciferase reporter plasmid (Addgene), using JetPRIME transfection reagent (Polyplus). Following 24 h the medium was changed to serum‐free medium containing ITS and stimulated for a further 24 h before collection and analysis using Dual luciferase reporter assay system (Promega).

### 2.4 In vitro micromass culture and alcian blue staining for functional analysis

Human articular chondrocytes (< 6 population doublings), were plated in dense cultures (2e7cells/ml) to allow matrix deposition. Experiments were conducted in serum‐free medium containing ITS, over a period of 6 days. For IL‐1β experiments, micromasses were cultured for 4 days in serum‐free medium and stimulus added for the final 2 days. Micromasses were then fixed with methanol at ‐20 then stained with 0.5% Alcian blue 8GS (Carl Roth C.l. 74240), over‐night. The following day the dye was extracted using 8 M Guanidine HCL and the proteoglycan content quantified using a spectrophotometer (De Bari et al., 2001). To account for any cell proliferation, total proteoglycans were normalized for DNA content.

### 2.5 QPCR for functional analysis

Human articular chondrocytes (< 6 population doublings), were plated in monolayer and stimulated under serum‐free conditions for 24 h. RNA was extracted from cells using TRIzolTM reagent (Invitrogen 15596026), cDNA was then synthesized using oligo(dt) primers (Promega C1101) and SuperscriptTM III Reverse transcriptase (Invitrogen 18080093). QPCR analysis was performed for the desired genes. Primer sequences are as follows: Actin 5′ TGACGGGGTCACCCACACTGTGCCCATCTA and 3′ CTAGAAGCATTTGCGGTGGACGATGGAGG, Human Sox9 5′ GAACGCACATCAAGACGGAG and 3′ TCTCGTTGATTTCGCTGCTC, Human Aggrecan 5′GTTGTCATCAGCACCAGCATC and 3′ACCACACAGTCCTCTCCAGC, Human Col2a1 5′CTGCTCGTCGCCGCTGTCCTT and 3′AAGGGTCCCAGGTTCTCCATC, Human MMP13 5′ ACGGACCCATACAGTTTGAATACAGC and 3′ CCATTTGTGGTGTGGGAAGTATCATC. Human MMP3 5′ CAACCGTGAGGAAAATCGATGCAG AND 3′ CGGCAAGATACAGATTCACGCTCAA, Human MMP8 5′GTCATTGTTTCCCATCACTGTATCCATT AND 3′ GGACACAATTCAACCCACGAAACA, Human PCNA 5′ GGAGAACTTGGAAATGGAAAC AND 3′ CTGCATTTAGAGTCAAGACCC.

### 2.6 In vivo models

The *TCF/Lef:H2B‐GFP* reporter line was donated by Anna‐Katerina Hadjantonakis, Memorial Sloan‐Kettering Cancer Center (Ferrer‐Vaquer et al., 2010). JAX stock #013752.

For analysis of joint tissue penetration by exosomes, up to 7 μl of exosome preparation or PBS vehicle was injected intra‐articularly in the joints of adult transgenic mice expressing green fluorescence protein (GFP) under the control of the Tcf/Lef promoter (*TCF/Lef:H2B‐GFP*) (Ferrer‐Vaquer et al., 2010).

For analysis of joint tissue repair, ten week old, male C57BL/6 mice were anesthetized with isofluorane. A cylindrical defect (0.78+/‐ 0.042 mm wide and 1.79+/‐ 0.056 mm deep) was generated in the lateral femoral condyle using a 21gauge needle (Thomas, Eldridge et al. manuscript in preparation). Liquid rat collagen type 1 gel containing exosomes was injected using a pulled glass pipette tip with a diameter of approximately 10 μm mounted at the end of a regular 2 μl pipette tip until the defect was full. The gel solidifies at 37°C. The joint was closed with an interrupted suture. After recovery mice were left free to move and feed ad libitum in filter cages. The operator and the scorers were blind to the treatment. The animals were monitored for signs of suffering and local infection at least weekly. Mice were killed 8 weeks after injury and the joints taken for analysis.

### 2.7 Staining and immunofluorescence

Following dissection, mouse knees were fixed overnight with buffered 4% paraformaldehyde, then decalcified for 24 h in formic acid buffer (33% formic acid, 13.5% trisodium citrate) at room temperature. Joints were than washed with water and embedded in OCT for cryosectioning (at 5μM). For proteoglycan analysis, sections were stained with 0.2% Safranin O in acetate buffer pH4. Safranin‐O intensity was measured by densitometry analysis, performed using ImageJ software as previously described (Nalesso et al., 2016). For immunofluorescence staining, sections were probed with anti‐GFP antibody (Abcam ab290). Percentage GFP positive cells were calculated as the percentage of fluorescent cells over the total number of cells present in the area analysed. The same approach was applied when quantifying the percentage of Ki67 positive cells, which were fixed with 4% paraformaldehyde and stained with Ki67 antibody (Abcam 15580) and dapi.

### 2.8 Ethics

Animal experiments were conducted under Home Office license 70/7986. Human samples were approved and obtained under the East London and the city research ethics commitee3 (Rec N.07/Q0605/29). Adult human articular cartilage was obtained from patients undergoing joint replacement for knee OA after obtaining informed consent.

### 2.9 Statistical analysis

Parametric data were compared with the t‐test, non‐parametric data with the Mann–Whitney test. Paired analysis was used for in vivo comparisons, where controls were contralateral knees. For multiple comparisons, an ANOVA or Kruskal‐Wallis, including the Tukey's post‐test, was used. *P* ‐Values < 0.05 were considered significant: *P < 0.05;** *P* < 0.01;*** *P* < 0.001.

## 3 RESULTS

### 3.1 WNT3a inhibits specific functions of IL‐1β in chondrocytes

**FIGURE 1:**
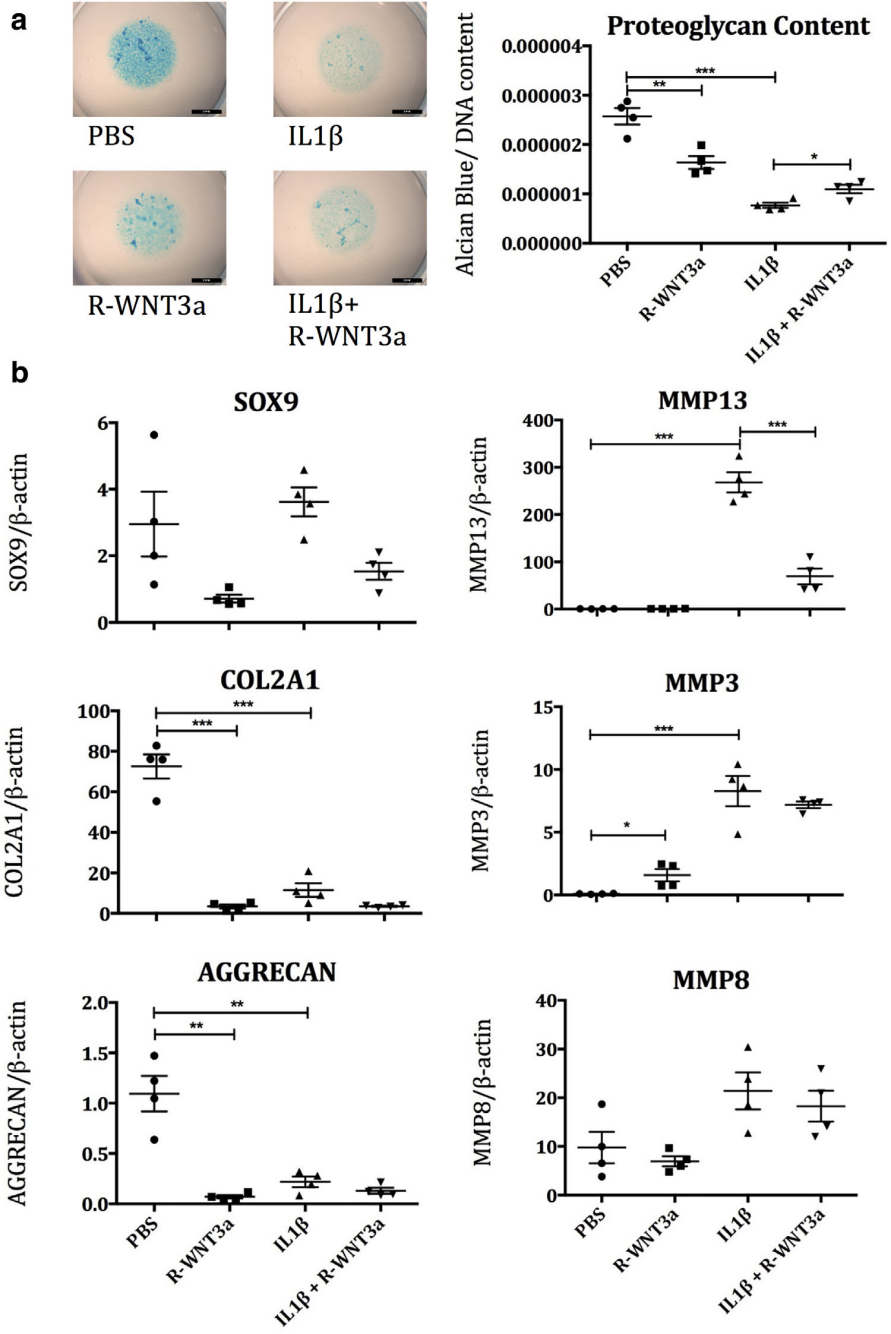
a) HAC micromasses were treated with combinations of recombinant WNT3a (50 ng/ml R‐WNT3a) and IL1‐β (10 ng/ml) over 6 days, then stained with alcian blue (Ab) dye to assess proteoglycan content, which was normalised to DNA content to account for proliferation (n = 4). b) HAC were treated with IL1‐β (10 ng/ml) and R‐WNT3a (100 ng/ml) and mRNA readouts of cartilage anabolism and IL1‐β pathway activation assessed by QPCR after 24 h (n = 4)

Excessive activation of WNT‐β‐catenin signalling results in loss of cartilage extracellular matrix; nevertheless, WNT3a expression at the injury site facilitates repair (Cheverud et al., 2014). We hypothesized that WNT3a may result in different outcomes in chondrocytes, depending on whether they are in resting conditions or during conditions of challenge. To test this hypothesis we assessed the effect of recombinant WNT3a (R‐WNT3a), on cartilage catabolism in resting chondrocytes and in the presence of an inflammatory stimulus, Interleukin‐1β (IL‐1β). As expected, R‐WNT3a treatment of human articular chondrocytes (HAC), in micromass culture, resulted in a reduction in proteoglycan content (Figure 1a). IL‐1β treatment decreased proteoglycan content to an even greater extent, however co‐treatment with WNT3a lead to a partial rescue of IL‐1β ‐induced proteoglycan loss (Figure 1a). In keeping with this, R‐WNT3a like IL‐1β caused a loss of chondrocyte markers *COL2a1* and *AGGRECAN*, as well master transcription regulator of cartilage, *SOX9*, but was able to inhibit matrix‐metalloprotease‐13 (*MMP‐13*) upregulation caused by IL‐1β. Under these experimental conditions however, WNT3a did not show the same effect on other MMPs upregulated by IL‐1β such as MMP‐3 and MMP‐8 (Figure 1b). These results encouraged us to explore the use of WNT3a to support cartilage repair.

### 3.2 WNT3a is transferred and carried on exosomes

**FIGURE 2:**
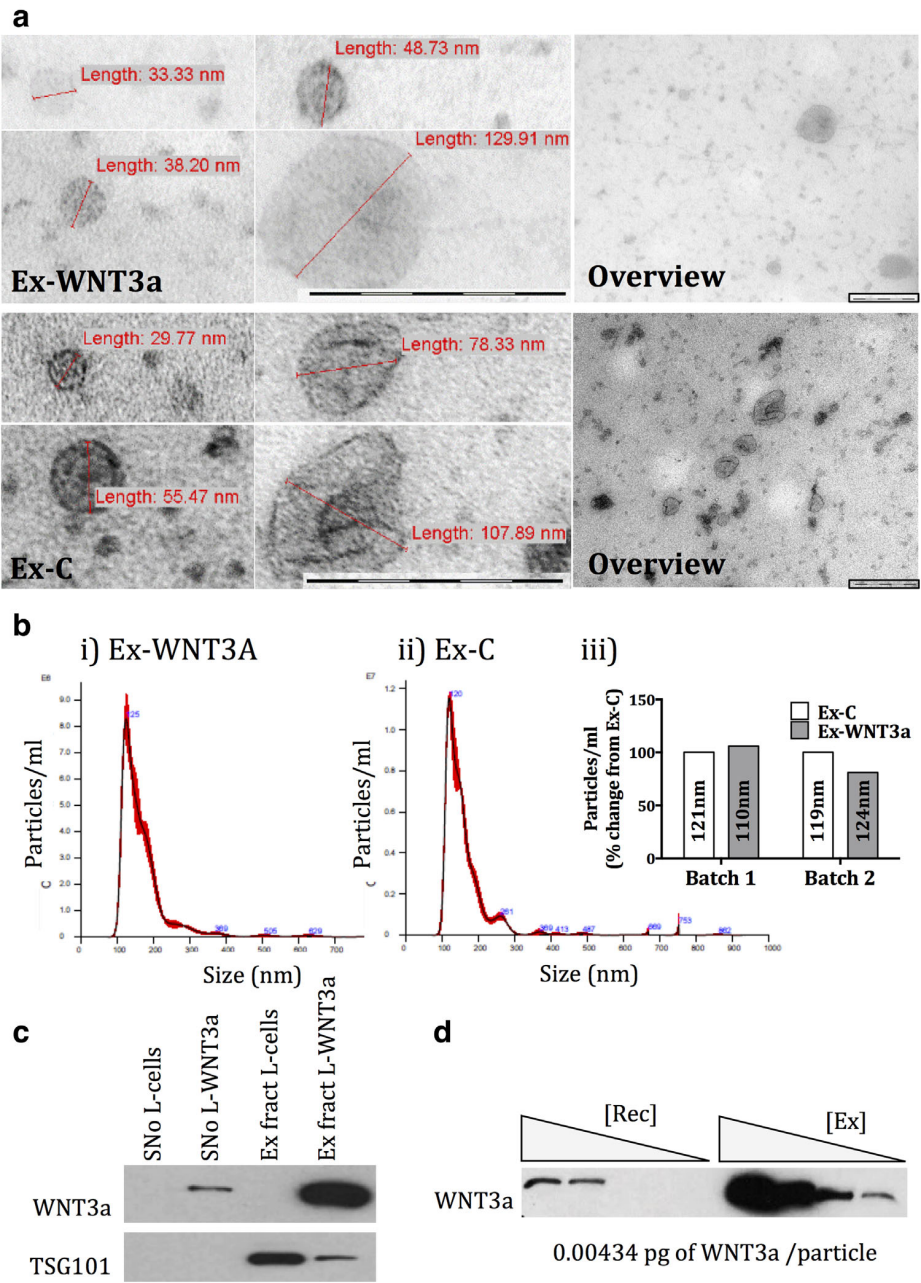
a) Electron microscopy of Ex‐WNT3a and Ex‐C. Includes an overview and zoomed in images. Scale bars on all images represent 0.2 μm. b i and ii) Nanoparticle tracking analysis of representative Ex‐WNT3a and Ex‐C preparations using NanosightTM(NS300). b iii) Comparison of number of particles and modal size of the particles (nm) for both Ex‐C and Ex‐ENT3a across different batches. c) Western blot for WNT3a and Tsg101 (exosomal marker) of 20 ul of supernatant (SNo) and exosome pellet/fraction (Ex fract) of L‐WNT3a and control L‐cells, following 100,000 × *g* ultracentrifugation (n = 2). d) Western blot for WNT3a using a standard curve of R‐WNT3a [Rec] and varying amounts of Ex‐WNT3a [Ex] to determine concentration of WNT3a in exosome preparation (n = 3). Detailed explanation in supplementary Figure S1

WNTs are palmitoylated, rendering them insoluble (Willert et al., 2003). In order to overcome this, and to allow long‐distance signalling, endogenous WNTs are secreted from cells on the surface of exosomes or associated with other carrier molecules such as SWIM (Gross et al., 2012). Since we previously demonstrated that extracellular vesicles actively enter the cartilage matrix (Headland et al., 2015), we tested whether we could use extracellular vesicles to deliver WNT3a to chondrocytes. This was facilitated by the fact that overexpressed WNT3a is spontaneously assembled and secreted on the external surface of exosomes as previously demonstrated by electron microscopy and immunogold labelling (Gross et al., 2012, Koch et al., 2014). WNT3a‐containing exosomes (Ex‐WNT3a) or control exosomes (Ex‐C) were purified by ultracentrifugation from conditioned medium generated from well‐established cell lines, L‐cells stably expressing WNT3a (L‐WNT3a) (Gross et al., 2012), or from control L‐cells (Figure 2). Exosomes had the expected shape and size distribution and no major differences were observed between the two populations, as assessed by electron microscopy and nanoparticle tracking analysis (Figure 2a+b). Although the number of exosome batches produced was too low to allow proper statistical analysis, we did not observe any differences in size or the ratio of exosomes produced between treatments or across different batches of exosomes, generated at different times (Figure 2b iii). The exosome preparation contained the exosomal marker TSG101 (Figure 2c) (Gross et al., 2012). WNT3a could be detected on exosomes generated from L‐WNT3a cells but not on those generated from control L‐cells (Figure 2c). By comparison with a standard curve generated with known amounts of R‐WNT3a, we could estimate that the concentration of WNT3a in the exosomal preparation was 0.0043 pg/particle (Figure 2d and Supplementary FIGURE S1). An equal number of Ex‐c and Ex‐WN3a particles were compared in all functional assays.

### 3.3 Exosomes can deliver functionally active WNT3a into cartilage in vitro

**FIGURE 3:**
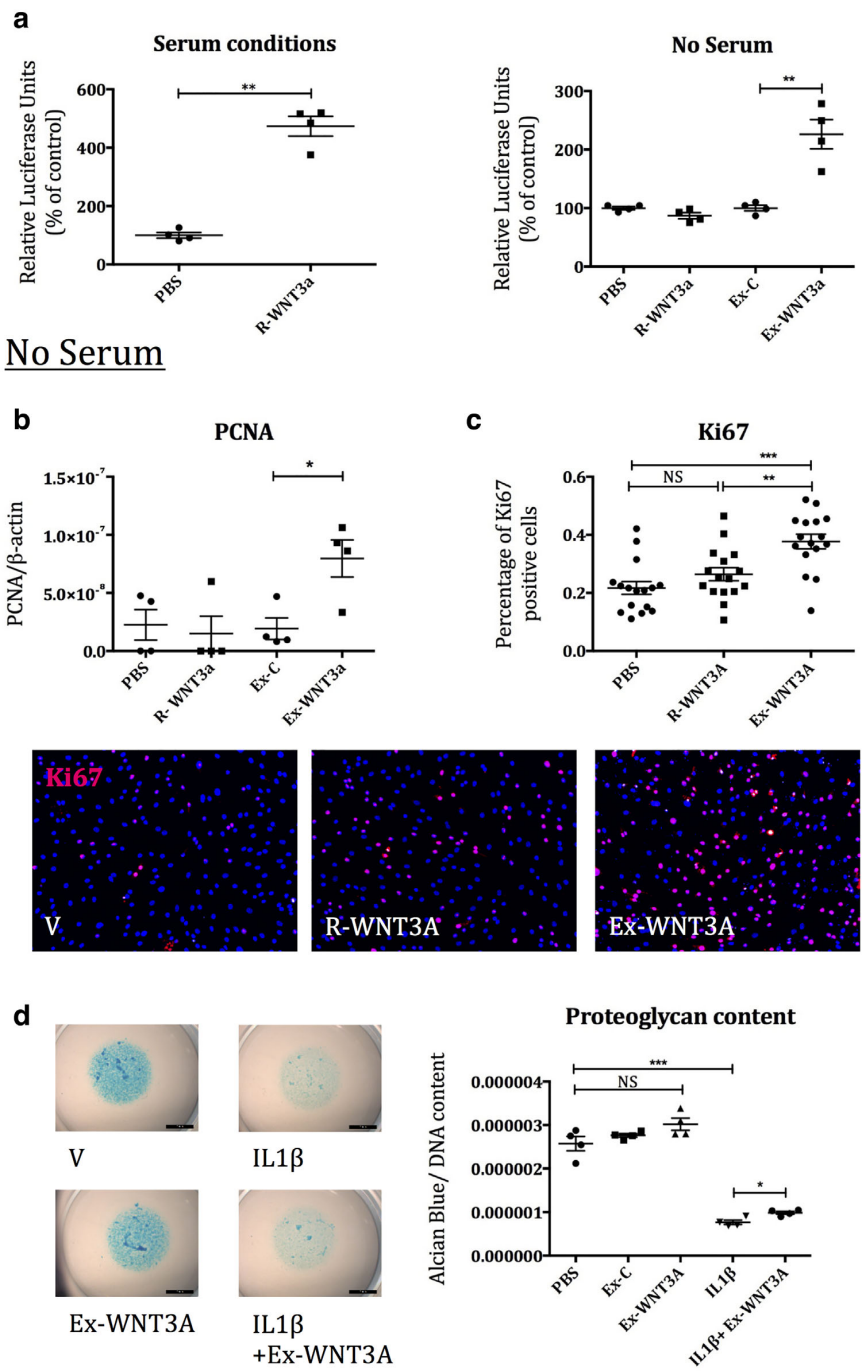
a) SUPER8TOPFlash reporter assay in HEK293 cells comparing R‐WNT3a and Ex‐WNT3a, both serum and serum‐free conditions (estimated 100 ng/ml WNT3a, n = 4). b) HAC were treated with Ex‐WNT3a, Ex‐C, R‐WNT3a or PBS, for 24 h in serum‐free conditions (estimated 100 ng/ml WNT3a). QPCR for PCNA mRNA was conducted (n = 4). c) HAC from two separate donors, were treated with Ex‐WNT3a (50 ng/ml) and equal concentration of R‐WNT3a for 24 h in serum‐free conditions. Cells were stained for Ki67 protein to assess cell proliferation (n = 8 per donor). Representative pictures for Ki67 analysis shown below. d) HAC micromasses were treated with combinations of Ex‐WNT3a (50 ng WNT3a protein) and IL1‐β (10 ng/ml) over 6 days in serum‐free conditions, then stained with alcian blue dye to assess proteoglycan content (n = 4)

Ex‐WNT3a for in vitro experiments were administered in order to dose either an estimated 100 ng/ml or 50 ng/ml of exosome associated WNT3a for comparison against the same concentration of recombinant WNT3a. To assess whether WNT3a was expressed on exosomes in an active form, we tested whether Ex‐WNT3a could activate the WNT‐β‐catenin dependent SUPER8TOPFlash reporter assay in HEK293 cells. These experiments showed, not only that Ex‐WNT3a efficiently activated the reporter assay, but that Ex‐WNT3a activity was maintained also in serum‐free conditions. Whereas R‐WNT3a was inactive in the absence of serum, presumably due to its poor solubility (Willert et al., 2003) (Figure 3a). This suggested that exosomes are efficient carriers for WNTs and deliver them in a format that enables signalling. Next we tested whether the exosomal delivery of WNT3a modulates its functional activity. We chose the following biological outcomes to test: chondrocyte proliferation and reduction of proteoglycan content (Nalesso et al., 2011 *]*. In vitro, Ex‐WNT3a upregulated markers of proliferation, including *PCNA* mRNA and Ki67 protein (Figure 3b+C). Importantly, Ex‐WNT3a induced greater proliferation than an equivalent amount of the R‐WNT3a. This further confirms results seen in Figure 3a, that WNT molecules do not signal efficiently in the absence of serum, but transport on exosomes is able to overcome this problem. To validate that Ex‐WNT3a had the same protective effects as R‐WNT3a (Figure 1a), we treated HAC micromasses with IL‐1β and/or equal concentration of Ex‐WNT3a. Interestingly Ex‐WNT3a alone, did not induce loss of proteoglycan of the HAC micromass (Figure 3d). This is in contrast with that seen for R‐WNT3a (Figure 1a). When administered in combination with IL‐1β however, which caused a decrease in proteoglycan content, similarly to the recombinant protein, Ex‐WNT3a resulted in a slight but significant rescue (Figure 3d).

### 3.4 Exosomes can penetrate cartilage tissue and deliver biologically active WNT3a in vivo

**FIGURE 4:**
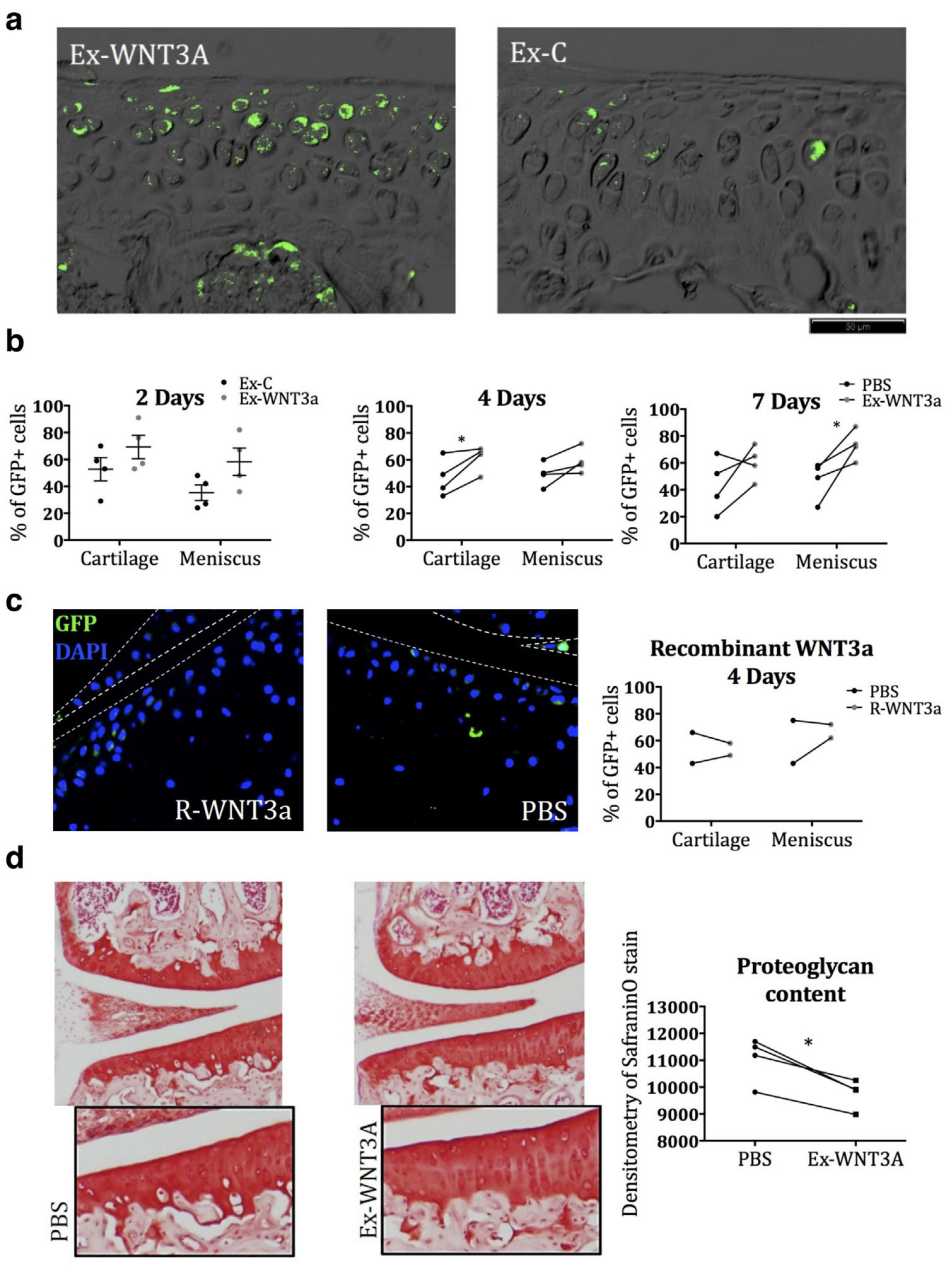
a) Immunofluorescence for GFP in TCF/Lef:H2B‐GFP reporter mice injected intra‐articularly with Ex‐WNT3a or Ex‐C (representative images from the 2 day end point). b) Quantification of immunofluorescence for GFP in TCF/Lef:H2B‐GFP mice injected with Ex‐WNT3a or control and killed after 2, 4 and 7 days (n = 4 per time point). For the 2‐day end point, Ex‐WNT3a containing 18 ng of WNT3a was compared to an equal particle number of Ex‐C (4.2e6 particles). In the 4 and 7‐day end point, Ex‐WNT3a containing 44 ng of WNT3a (10.1e6 particles) was compared to contralateral control knees injected with PBS vehicle. c) Additional reporter mice were injected with 44 ng of WNT3a recombinant and compared to their contralateral controls. d) Safranin‐O staining of mice injected with Ex‐WNT3a (containing 44 ng of WNT3a) or PBS and killed 7 days later (n = 4)

To assess whether exosomes could deliver active WNT3a into the cartilage matrix and signal to chondrocytes in vivo we used a TCF/Lef:H2B‐GFP transgenic mouse line. These mice express green fluorescence protein (GFP) under the control of the Tcf/Lef promoter (TCF/Lef:H2B‐GFP)[20]. Since Tcf/Lef is a sensitive target of WNT‐β‐catenin signalling, GFP becomes expressed in cells in which signalling is activated. As WNT3a is a potent activator of the WNT‐β‐catenin pathway, a strong induction of GFP was expected upon WNT3a delivery. Stimulus was administered intra‐articularly, to ensure that exosomes reached the cartilage within the joint. The joint cavity has a relatively low volume and therefore limits the amount of liquid that can be injected (up to 7 μl). We administered the maximum volume possible to ensure sufficient concentrations of WNT3a, which dictated the number of exosomes particles administered. This resulted in the administration of an estimated 18 ng of WNT3a (4.2e6 particles for both Ex‐WNT3a and Ex‐C) at the 2 day end point, and 44 ng of WNT3a (10.1e6 particles of Ex‐WNT3a compared to PBS vehicle) at the 4 and 7 day endpoints. 44 ng of recombinant WNT3a was administered. In vivo, the intra‐articular injection of Ex‐WNT3a in TCF/Lef:H2B‐GFP reporter mice (Figure 4a) resulted in the activation of WNT‐β‐catenin signalling within the full thickness of the articular cartilage and menisci as assessed by immunofluorescence for GFP (Figure 4b). Importantly the same concentration of R‐WNT3a, failed to activate the GFP reporter (Figure 4c). In keeping with the known capacity of WNT3a to reduce glycosaminoglycan in cartilage tissue (Nalesso et al., 2011), Safranin‐O staining was reduced in the cartilage of mice treated with Ex‐WNT3a, 7 days post injection (Figure 4d).

### 3.5 Exosomes loaded with WNT3a improved osteochondral repair in mice

**FIGURE 5:**
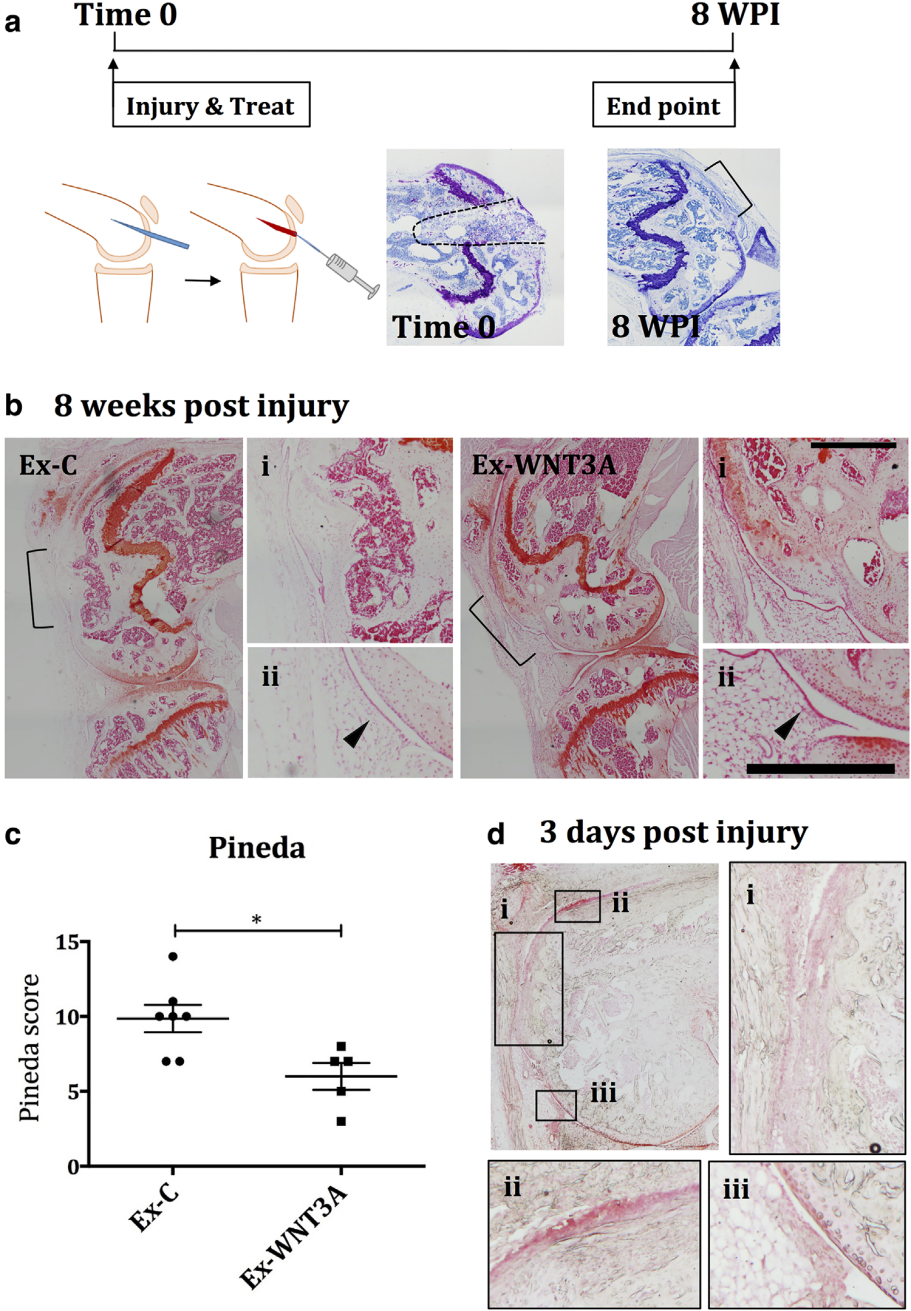
An osteochondral defect was generated in the lateral femoral condyle and filled with 2 μl of rat collagen type1 gel, containing treatment. a) Scheme of joint injury model, including images of time 0 and 8 weeks post injury (WPI) without treatment. b) Defects were treated with either Ex‐WNT3a (estimated 5 ng WNT3a protein) or Ex‐C (equivalent number of particles, 1.2e6 particles per knee) at time 0 and the experiment was terminated at 8 WPI (n = 7 for Ex‐C and n = 5 for Ex‐WNT3a). Knees were sectioned and stained with Safranin‐O. Defect margins are depicted by black brackets in over view images. Higher magnification images are shown in i (defect area at cartilage surface) and ii (synovium), with black arrows indicating synovial lining. c) Repair was assessed using the Pineda score at 8 WPI. d) Additional mice were treated exactly as in the 8 week experiment and instead terminated at 3 days post injury to assess the early phases of repair. Images show an overview of the injured joint and insets at a higher magnification: i) showing the cartilage surface in the area of the defect, ii) showing fibrillation on the cartilage surface away from the defect, and iii) showing proliferation in the synovial lining

WNT3a is genetically associated with the capacity to repair the ear lobe in mice (Cheverud et al., 2014). To investigate if Ex‐WNT3a enhanced the repair of osteochondral defects in the knee, we generated critical‐size osteochondral defects on the lateral femoral condyle of adult mice and filled it with rat collagen type1 gel containing either Ex‐WNT3a or Ex‐C (Figure 5a). As with previous in vivo investigations (Figure 4) we administered the maximum number of particles possible into the volume of the defect, resulting in an estimated 5 ng of WNT3a delivered (1.2e6 particles for both Ex‐C and Ex‐WNT3a). Eight weeks later, joints treated with Ex‐WNT3a had an improved repair score (measured by the Pineda score (Pineda et al., 1992)), when compared to joints treated with Ex‐C (Figure 5b+C). Histological images show an overview of the joint (Figure 5b), a higher magnification of the cartilage surface in the region of the defect (Figure 5b i), and a higher magnification of the synovial lining (Figure 5b ii), which at this late endpoint, showed no hyperplasia/ thickening of the synovial lining. In a second experiment, we repeated the defect and treatment regime but this time terminated at only 3 days post injury to assess the situation in the joints during the early phases of repair. No visible histological differences were observed at this stage, between joints treated with either Ex‐C or Ex‐WNT3a (representative images shown in supplementary Figure S2). However cartilage surface/joint damage, consistent with the early phases following injury, were observed in all joints and are represented in Figure 5d. Histological images show an overview of an injured knee joint, with insets showing damaged cartilage/ subchondral bone tissues in the outer regions of the defect (Figure 5 i), cartilage surface fibrillation in regions of the joint away from the defect (Figure 5 ii), and thickening of the synovial lining (Figure 5 iii), consistent with the expected inflammation present at this early stage following injury.

## 4 DISCUSSION

We have shown that the use of exosomes as a vehicle enables the delivery of WNT3a into the avascular cartilage in a biologically active form and promotes the healing of critical size osteochondral defects in mice.

Chondrocytes are encased in a dense, negatively charged, avascular extracellular matrix, which is poorly permeable to macromolecules. For instance, antibodies do not penetrate the cartilage extracellular matrix (Maroudas, 1976) and even antigen binding fragments only enter the very superficial layers (Byun et al., 2013). In addition, WNT molecules are relatively insoluble (Fuerer et al., 2010) and bind to heparansulphate proteoglycans which can further reduce their passive diffusion into cartilage. In view of this, it is not surprising that the injection of recombinant WNT3a did not elicit a signalling response.

In contrast, we have provided evidence that exosomes achieve efficient and prolonged delivery of this particularly challenging molecule, into the full depth of the cartilage. This represents a technological breakthrough for several molecular targets that have been validated using transgenic approaches in repair as well as in osteoarthritis (Araldi et al., 2011; Glasson et al., 2005; Johnson et al., 2012; Kim et al., 2014; Monteagudo et al., 2017; Sampson et al., 2011; Xie et al., 2010, 2016; Zhen et al., 2013), but where the efficient delivery of a recombinant protein is an obstacle towards clinical application.

Extracellular vesicles have the advantage over synthetic carriers such as liposomes or capsules in that they have active mechanisms of motility and homing. For instance, extracellular vesicles, but not capsules of the same size could penetrate the avascular cartilage extracellular matrix and reach chondrocytes (Headland et al., 2015). In recent years the potential of exosomes as delivery vehicles has been well recognized and interest from the pharmaceutical industry has soared resulting in technological advancements in the field. These include breakthroughs in loading and delivery of cargo (Heath et al., 2019) as well as improvements and up scaling for production and purification. In this study we chose ultracentrifugation for exosome preparation, this is in adherence with previous reports studying the secretion of WNTs on exosomes (Gross et al., 2012; Koch et al., 2014). Although widely used, when considering large scale production and preparation, limitations such as the co‐isolation of non EV materiel and the tedious nature of the technique for low recovery of product, makes the method unsuitable. Novel and improved technologies, will therefore be essential when considering exosomes for therapy. In addition to production, challenges in bringing these novel EV based therapeutics to the clinic will also need to be overcome. It is likely that given their cellular origin and complex composition, they will require an approach similar to that of cell based therapies (Lener et al., 2015). Never the less, utilizing and expanding on this opportunity to overcome challenges in delivering therapy to cartilage, in the future could lead to safe and efficient production of exosomes from validated cell lines for delivery of even large complicated molecules, thus removing one of the biggest challenges in treating cartilage injury.

We previously showed that extracellular vesicles actively penetrate the cartilage extracellular matrix (Headland et al., 2015). It is not clear whether this active penetration is due to adhesion properties and motility or whether the extracellular vesicles follow chemotactic cues. Here we showed the successful delivery of a WNT3a signal by exosomes, to chondrocytes within their extracellular matrix, however, we did not demonstrate that the exosomes physically enter the matrix and reach the chondrocyte cell membrane. It is therefore also possible that WNTs are released from the exosomes before entering cartilage or that they trigger relay mechanisms. The delivery of WNT3a on exosomes led to a surprisingly long‐lasting signalling response, suggesting that the exosomes not only conferred WNT3a signal into the cartilage, but also may prolong the local half‐life.

It is important to highlight how a single delivery of exosomal WNT3a was sufficient to improve repair, which was evident 8 weeks later. We did not observe any differences between Ex‐WNT3a and Ex‐C treated knees at 3 days post injury, and therefore, cannot confirm a precise mechanism of action for Ex‐WNT3a in joint repair. It is possible that the huge endogenous response occurring in the joint following injury, could mask differences between treatments. Considering the biology of WNT3a, and our WNT pathway activation data in healthy mouse knees (Figure 4), we can hypothesize that the long‐lasting effect was due to the retention of WNT3a in an active form for prolonged or increased signalling. It is also possible that a relatively short signalling event by WNT3a may have triggered homeostatic effects that are self‐maintained or lead to long‐term outcomes. This is suggested by the fact that a single, short episode of activation of β‐catenin led to the formation of thicker cartilage in mice (Yuasa et al., 2009).

Whereas there is abundant literature covering the genetics of osteoarthritis, the genetics of repair are understudied. A recent publication identified a single nucleotide polymorphism in the Wnt3a locus which was associated with the capacity of mouse sub‐strains to repair punch‐holes in the ear pinna (Cheverud et al., 2014). In keeping with a direct function in repair mechanism, WNT3a protein was more strongly expressed at the healing edge of the ear cartilage in mouse strains that healed better. This, and the capacity of WNT3a to profoundly modulate the chondrocyte phenotype (Nalesso et al., 2011) motivated our choice for this target.

Our data show that WNT3a was able to drive an improved repair following joint injury (Figure 5). In spite of this, and the genetic evidence supporting a positive role of WNT3a in cartilage repair and its well‐known capacity to support cartilage stem cells (Yasuhara et al., 2011), WNT3a is also known to mediate catabolic functions such as loss of extracellular matrix components (Figure 1 and reference (Nalesso et al., 2011)). This apparent paradox is explained by the fact that joint surface repair requires a sequence of events in a precise, tightly regulated temporal order. The early phases immediately after injury are characterized by expansion of stem cell populations (Dell'accio & Vincent, 2010; Roelofs et al., 2017) and extracellular matrix remodelling (Dell'accio & Vincent, 2010; Dell'accio et al., 2008; Sherwood et al., 2014). The late phases, instead, are characterized by tissue patterning followed by extracellular matrix synthesis and maturation (Eltawil et al., 2009). The choice of therapeutic agents to enhance repair, therefore, will need to be targeted to the specific repair phase. In this context, we chose to deliver WNT3a in the early phase of repair when matrix remodelling and cell proliferation are required to replace damaged cells and tissues, rather than in the later phases when WNT3a may result in detrimental effects such as loss of proteoglycans. In healthy cartilage WNT signalling needs to be maintained to a minimum, whereas cartilage injury triggers a transient wave of WNT activation (Dell'accio et al., 2006, 2008) which is required to support local progenitor cells (Yuasa et al., 2009). Therefore, while administering WNT3a to recently injured cartilage reinforced a naturally occurring repair mechanism which ultimately led to increased proteoglycan content, its administration in healthy cartilage triggered an early, likely transient, matrix remodelling, which would be useful during the early phases of repair, but is undesirable in homeostatic conditions.

In keeping with this observation, in our experiments, recombinant WNT3a had a dramatically different effect in healthy cartilage (in which it drove the loss of extracellular matrix), compared to inflammatory conditions (in which it reduced the capacity of IL‐1β to cause loss of proteoglycans) (Figure 1). Interestingly, unlike recombinant WNT3A, WNT3A associated to exosomes did not result in proteoglycan loss in chondrocyte micromasses (Figure 3d). While we do not have experimental data do explain this discrepancy, it is possible that the context of WNT3a presentation when assembled on exosomes may modify its affinity to its receptors and bias its signalling properties. Alternatively, the bioavailability of WNT3a assembled on exosomes may be different or be associated with different temporal dynamics compared to the naked recombinant protein.

Although, in our murine injury model, we could measure a statistically significant improvement of the repair outcome, the quality of the healed cartilage was not optimal. This could be due to different factors, including the size of the defect, which might be too large to achieve full repair, the dose of WNT3a, the time and frequency of administration, and the choice of the bioactive molecule being delivered. In this context, although our choice of WNT3a was justified by genetic evidence, other WNTs such as WNT16, which supports production of the joint lubricant, Lubricin (Nalesso et al., 2016), have shown perhaps a better pharmacological profile. Nevertheless, this study represents a unique proof of concept that supporting the early phases of repair has long term beneficial outcomes. This study also represents a proof of concept for this technology. The choice of the ligand, the generation of appropriate cell lines for exosome generation, and safe, optimal, consistent delivery, will be paramount for the clinical application.

In this study we have demonstrated that a single administration of WNT3a, a molecule upregulated by acute cartilage injury, enhances the long‐term outcome of cartilage repair. This highlights the importance of the early phases of injury response in priming the repair cascade. In addition, we have demonstrated that exosomes are able to deliver functionally active molecules into joint tissues, resulting in long lasting and protective effects. This presents a unique opportunity to harness the natural targeting power of exosomes for the benefit of therapy delivery across medicine.

## AUTHOR CONTRIBUTIONS

Francesco Dell'Accio and Bethan L. Thomas incepted the study. Bethan L. Thomas, Francesco Dell'Accio, Suzanne E. Eldridge, Ray Jupp, Adrian Moore, Alison Maloney, Giovanna Nalesso, Suzanne E. Eldridge, Anne‐Sophie Thorup, Sara Caxaria and Costantino Pitzalis contributed to experimental design. Bethan L. Thomas, Francesco Dell'Accio, Suzanne E. Eldridge, Babak Nosrati, Mario Alvarez, Anne‐Sophie Thorup, Sara Caxaria, Aida Barawi, James G. Nicholson performed the experimental work. Bethan L. Thomas, Francesco Dell'Accio, Ray Jupp, Adrian Moore, Alison Maloney, Anne‐Sophie Thorup, Sara Caxaria, Suzanne E. Eldridge and Carles Gaston‐Massuet interpreted results. Bethan L. Thomas, Francesco Dell'Accio, Alison Maloney, contributed to writing and completion of the manuscript. Mauro Perretti provided infrastructural support.

## COMPETING INTERESTS

The authors declare no conflict of interest

## Supporting information

Supporting Information

Supporting Information

Supporting Information
