# Supplementary figures and images for: WNT3A‐loaded exosomes enable cartilage repair
Source: J Extracell Vesicles. 2021 May 19;10(7):e12088. doi: 10.1002/jev2.12088 (PMC8134720; doi:10.1002/jev2.12088)

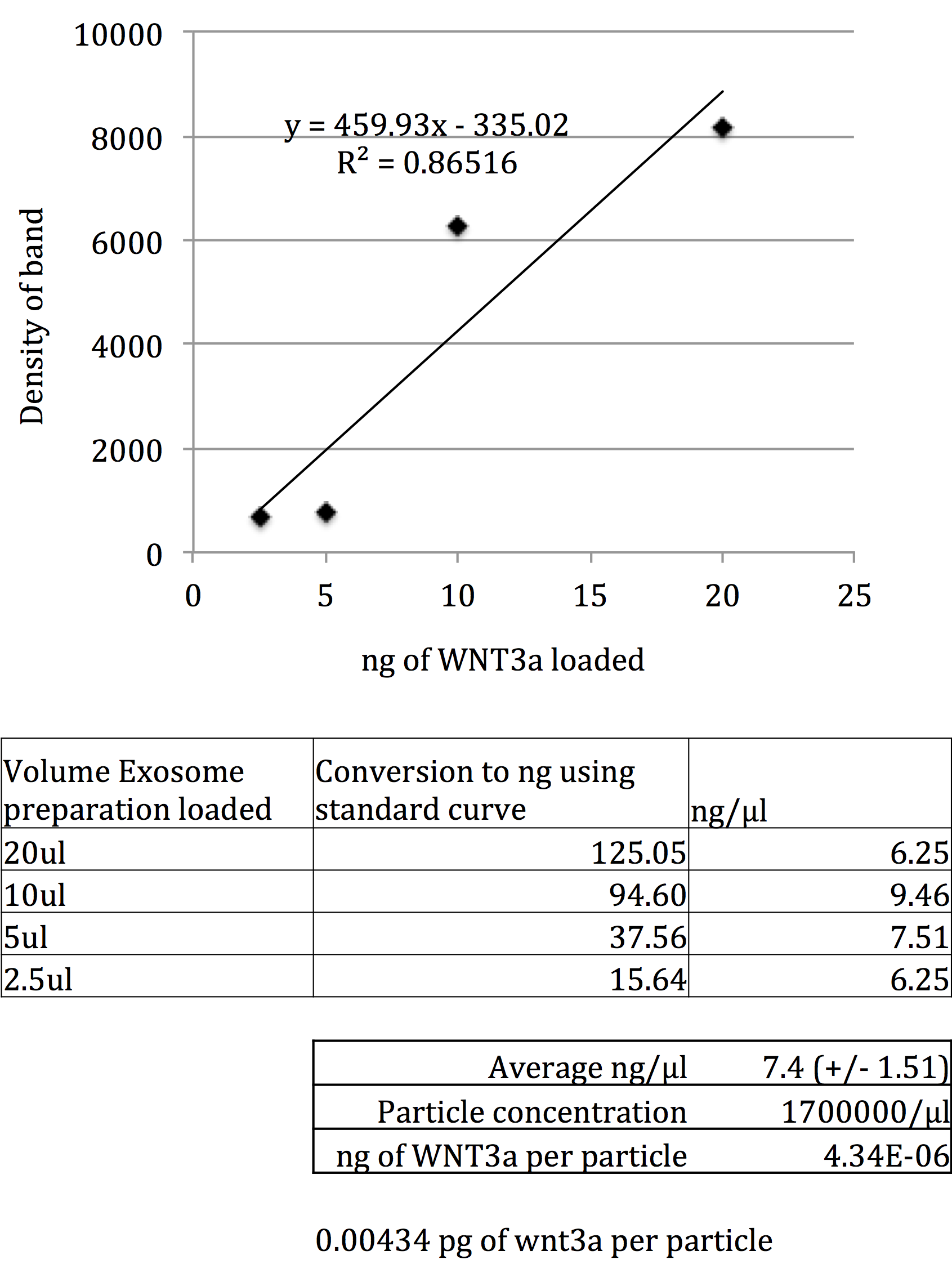

Supplement: Supplementary file 1 — Supporting Information [file JEV2-10-e12088-s003.tiff]

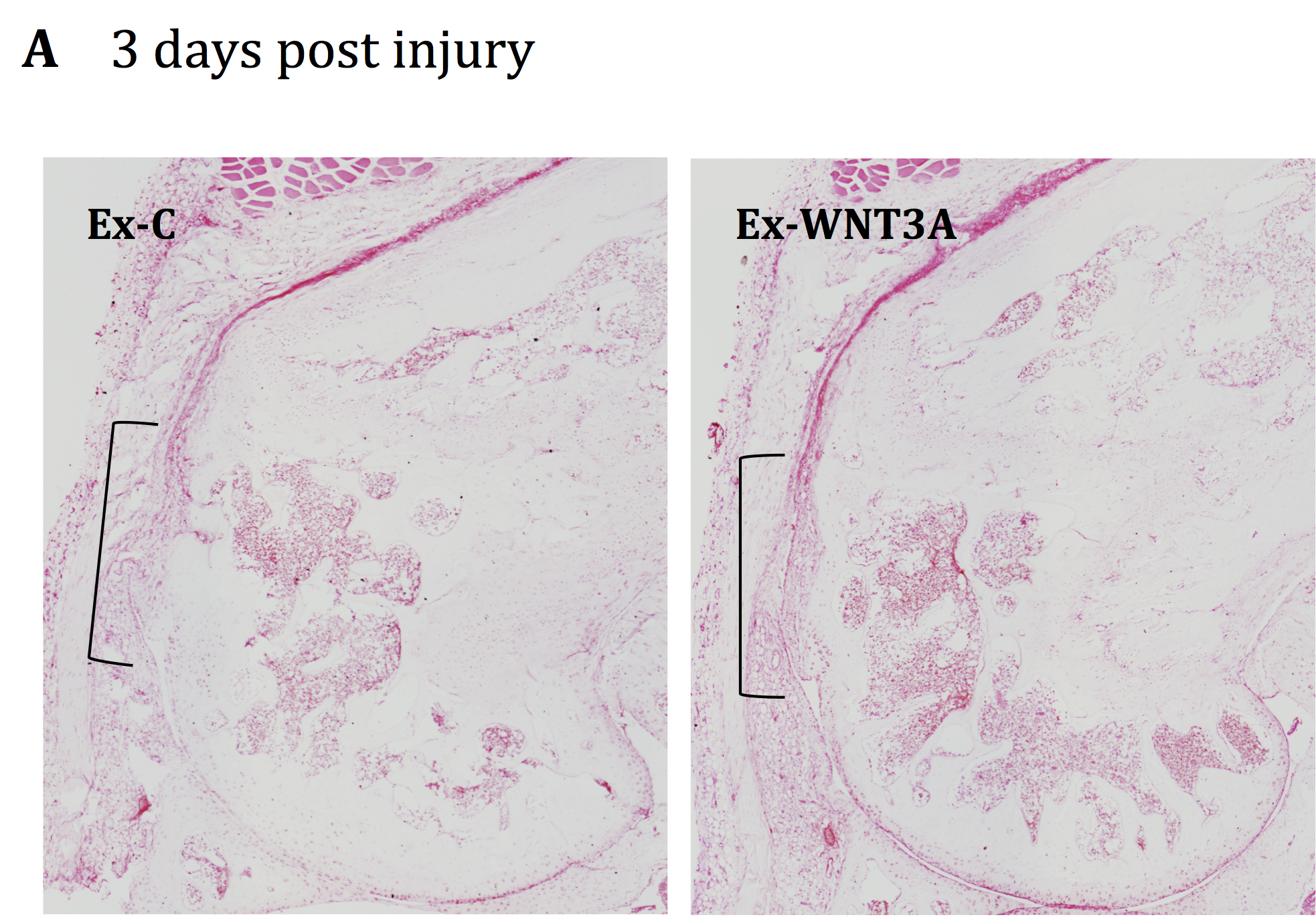

Supplement: Supplementary file 2 — Supporting Information [file JEV2-10-e12088-s002.tiff]
