# Supplementary material for: WNT3A‐loaded exosomes enable cartilage repair
Source: J Extracell Vesicles. 2021 May 19;10(7):e12088. doi: 10.1002/jev2.12088 (PMC8134720; doi:10.1002/jev2.12088)
Supplement: Supplementary file 3 — Supporting Information [file JEV2-10-e12088-s001.docx]

**SUPPLEMENTARY MATERIAL**

Supplementary figure 1: To calculate the concentration of WNT3a in the exosomes, different concentrations of R-WNT3a were run on a western blot and the band density was calculated to generate a standard curve. On the same blot, Ex-WNT3a was also run and the concentrations extrapolated using the standard curve. Three different exposures were generated for the blot and the most appropriate was selected for concentration analysis. Variance is represented as +/- standard deviation.

Supplementary figure 2: As in figure 5D, an osteochondral defect was generated in the lateral femoral condyle and filled with 2μl of rat collagen type1 gel, containing treatment, either Ex-C or Ex-WNT5a (estimated 5ng WNT5a protein). The experiment was terminated 3 days following injury and treatment, and knees sectioned for Safranin O staining. Black lines indicate the region of the defect within the knee.
